# Correction: Effects of water, ammonia and formic acid on HO_2_ + Cl reactions under atmospheric conditions: competition between a stepwise route and one elementary step

**DOI:** 10.1039/c9ra90058f

**Published:** 2019-07-29

**Authors:** Tianlei Zhang, Yongqi Zhang, Mingjie Wen, Zhuo Tang, Bo Long, Xiaohu Yu, Caibin Zhao, Wenliang Wang

**Affiliations:** Shaanxi Key Laboratory of Catalysis, School of Chemical & Environment Science, Shaanxi University of Technology Hanzhong Shaanxi 723001 P. R. China ztianlei88@l63.com +86-0916-2641083 +86-0916-2641083; School of Materials Science and Engineering, Guizhou Minzu University Guiyang 550025 P. R. China longbo@gzmu.edu.cn; Key Laboratory for Macromolecular Science of Shaanxi Province, School of Chemistry & Chemical Engineering, Shaanxi Normal University Xi’an Shaanxi 710062 P. R. China wlwang@snnu.edu.cn; Shanghai Key Laboratory of Molecular Catalysis and Innovative Materials, Fudan University Shanghai 200433 P. R. China

## Abstract

Correction for ‘Effects of water, ammonia and formic acid on HO_2_ + Cl reactions under atmospheric conditions: competition between a stepwise route and one elementary step’ by Tianlei Zhang *et al.*, *RSC Adv.*, 2019, **9**, 21544–21556.

The authors regret that incorrect details were given for ref. 52 in the original article. The correct version of ref. 52 is given below as ref. 1.

The Royal Society of Chemistry apologises for these errors and any consequent inconvenience to authors and readers.

## Supplementary Material
